# Sub-picosecond photon-efficient 3D imaging using single-photon sensors

**DOI:** 10.1038/s41598-018-35212-x

**Published:** 2018-12-07

**Authors:** Felix Heide, Steven Diamond, David B. Lindell, Gordon Wetzstein

**Affiliations:** Stanford University, Department of Electrical Engineering, Stanford, USA

## Abstract

Active 3D imaging systems have broad applications across disciplines, including biological imaging, remote sensing and robotics. Applications in these domains require fast acquisition times, high timing accuracy, and high detection sensitivity. Single-photon avalanche diodes (SPADs) have emerged as one of the most promising detector technologies to achieve all of these requirements. However, these detectors are plagued by measurement distortions known as pileup, which fundamentally limit their precision. In this work, we develop a probabilistic image formation model that accurately models pileup. We devise inverse methods to efficiently and robustly estimate scene depth and reflectance from recorded photon counts using the proposed model along with statistical priors. With this algorithm, we not only demonstrate improvements to timing accuracy by more than an order of magnitude compared to the state-of-the-art, but our approach is also the first to facilitate sub-picosecond-accurate, photon-efficient 3D imaging in practical scenarios where widely-varying photon counts are observed.

## Introduction

Active 3D imaging has broad applications across disciplines, including remote sensing, non-line-of-sight imaging, autonomous driving, robotics, and microscopic imaging of biological samples^1–11^. Key requirements for most of these applications include high timing accuracy, fast acquisition rates, wide dynamic operating ranges, and high detection sensitivity. In particular, remote sensing and automotive applications^1–4,6^ demand acquisition ranges from <1 meter to kilometers, while non-line-of-sight imaging^7–9^, for example, relies on the information encoded by the few returning photons of multiply scattered indirect light, in addition to the directly reflected light. To facilitate these applications, ultra-sensitive detectors have been developed that allow for individual photons returning from a pulsed illumination source to be recorded. One of the most sensitive time-resolved detector technologies that can be produced in the CMOS process to date are single-photon avalanche diodes (SPADs), which have been rapidly established as a core detector technology for 3D imaging^2,12–20^.

SPADs are reverse-biased photodiodes operated above their breakdown voltage, i.e. in Geiger mode^12^. Photons incident on the active surface of a SPAD can trigger an electron avalanche, which is subsequently time stamped. By repeatedly time stamping photons returning from a synchronously pulsed illumination source, typically operating at MHz rates, a histogram of photon counts over time can be accumulated. The histogram approximates the intensity of the returning light pulse and characterizes the propagation delay, enabling distance, reflectance and 3D geometry to be recovered. While a SPAD can be operated in a free-running mode^21^, which allows photon events to be detected at all arrival times simultaneously, some modes of operation enable gated detection wherein only photons within a specified time window between pulses are detected^22^. In this gated mode^23,24^, SPADs achieve high accuracy and robustness to ambient light by electronically gating out all but a narrow temporal slice^22^. However, gated acquisition requires a sequential sweep of the temporal slice over the full target range, which restricts it to applications that do not require fast acquisition rates. SPAD detectors operated in free-running mode do not suffer from this limitation, but whether these sensors are used for 3D imaging, microscopy, non-line-of-sight imaging, or any other application, they are subject to a fundamental phenomenon which severely limits accuracy: pileup distortions^25,26^.

Pileup is a limitation inherent to the SPAD detector’s working principle. After every triggered electron avalanche, the detector needs to be quenched before it is able to detect further photon arrival events. During this dead time, which is typically in the order of tens to hundreds of ns, the detector is inactive. Thus, earlier photons of a single laser pulse are more likely to trigger an avalanche while later ones are more likely to fall into the dead time, thus being ignored. This causes severe measurement skew, which is known as pileup. Pileup cannot be corrected in hardware and results in round-trip time-stamping errors that are orders of magnitudes larger than the timing precision of the detector. Note that the overall instrument dead time is the maximum of the detector dead time and the dead time of the time-to-digital conversion (TDC). However, TDC circuits with dead times of less than a nanosecond are available, such as the PicoQuandt TimeHarp Nano, which is one to two orders of magnitudes lower than typical detector dead times. Although this work is applicable to both TDC or detector-induced pileup, we consider detector-limited systems in the following.

**Figure 1:**
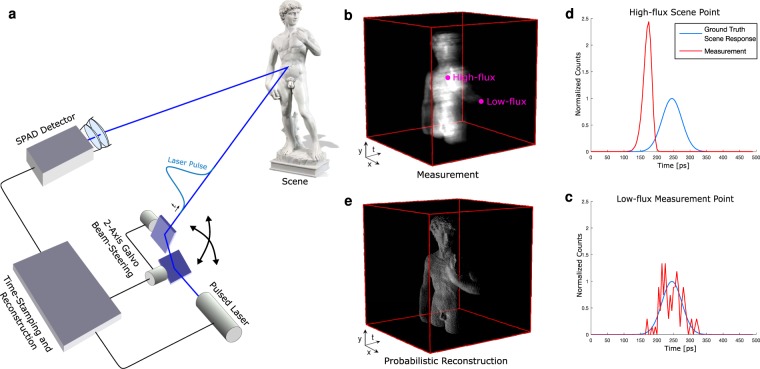
Sub-picosecond 3D Imaging Framework. (**a**) A collimated, pulsed laser illuminates the scene at a single point. The laser is laterally scanned using a 2-axis mirror galvanometer. Timing and control electronics time-stamp each detected photon arrival relative to the last emitted pulse and accumulate these events in a histogram of spatio-temporal photon counts **(b)**. This histogram is processed to estimate both reflectivity and depth information **(c)**. Two points are highlighted, one corresponding to high-flux **(d)** and the other to low-flux **(e)** measurements. Whereas the latter are noisy, high-flux measurements suffer from pileup distortion which introduce a significant bias for the depth estimation of conventional algorithms. The proposed estimation method accurately models both of these scenarios, allowing for reflectance information and travel time to be estimated with sub-picosecond accuracy from severely distorted measurements.

To avoid pileup distortion, active imaging systems can be operated in a low-flux regime, where the average number of incident photons per measurement is extremely low (i.e. ≪1). State-of-the-art depth and reflectivity estimation techniques have demonstrated successful operation in these challenging conditions using probabilistic image formation models and statistical priors in the inverse methods^18,27,28^. However, many 3D imaging applications, for example in robotics, biological imaging, or automotive sensing, operate in environments where both objects returning a low number of signal photons and objects reflecting higher numbers of photons are essential for decision making. The large variance in acquired photon counts of objects at different depths or resulting from varying reflectivity of different objects makes it crucial for a practical 3D imaging technique to operate robustly in both low-flux and high-flux conditions simultaneously (see Fig. 1).

In this work, we introduce a new estimation algorithm that overcomes existing limitations of active 3D imaging systems with free-running SPADs. Specifically, we demonstrate sub-picosecond timing accuracy for pulsed light sources with a full width at half maximum (FWHM) wider than 50 ps. The proposed method improves the accuracy of existing depth and albedo estimation algorithms by more than an order of magnitude across a wide dynamic range, from low-flux to high-flux measurements. These benefits are enabled by an image formation model and corresponding inverse method that lift assumptions of low-flux models^18,27^ to broad operating conditions that may be distorted by pileup. To this end, we introduce a probabilistic image formation model that includes pileup and we derive efficient inverse methods for the depth and albedo estimation problem with this model. The inverse methods exploit statistical priors of both depth and albedo. Unlike previous work, our reconstruction framework jointly estimates all unknown parameters, overcoming algorithmic limitations that restrict the timing precision of existing methods. The proposed methods not only facilitate highly accurate and fast 3D imaging but they also open up a new operating regime of photon-efficient 3D imaging in conditions with drastically-varying photon counts. Concurrently with our work, several other methods addressing the pileup problem for SPADs have recently been published^29,30^.

## Results

### Imaging setup

The experimental acquisition setup is illustrated in Fig. 1. The light source is a pulsed, collimated laser that is scanned horizontally and vertically using a 2-axis mirror galvanometer. Light scattered back from the scene is focused on the SPAD (Micro Photon Devices PD-100-CTD) using a microscope objective. A PicoQuant PicoHarp 300 module is used for time stamping, histogram accumulation, and synchronization of laser and detector. We use a pulse rate of 5 MHz, resulting in a maximum path length of 60 m without considering the instrumentation dead time. The dead time of the SPAD detector is 75 ns. Due to the fact that this dead time is multiple orders of magnitude larger than the desired timing accuracy, the pileup distortions of measured histograms can only be ignored in the ultra-low-flux regime with <10^−2^ average photons detected per pulse.

### Observation model

We discretize the scene into a grid of *m* × *n* points at distances ***z*** ∈ **R**^*m*×*n*^ and denote the reflectance of these points as ***α*** ∈ [0, 1]^*m*×*n*^. Each point is probed with *N* pulses.

We first describe the observation model for a single point (*i*, *j*) by modeling the temporal shape of the laser pulse *g* as a mixture of Gaussians

1 $$g(t)=\sum _{k=1}^{K}\,{a}_{k}\exp (-\frac{{(t-{b}_{k})}^{2}}{{c}_{k}^{2}}),$$

where the parameters *a*_*i*_, *b*_*i*_, *c*_*i*_ ∈ **R** are calibrated in a pre-processing step (cf. Methods). Given this parametric impulse model, the photon detections on the SPAD detector can be modeled as an inhomogeneous Poisson process with rate function

2 $${r}_{ij}(t,{\alpha }_{ij},{z}_{ij},s)=\mu {\alpha }_{ij}\,g(t-2{z}_{ij}/c)+s,$$

where *c* is the speed of light, *μ* is the SPAD photon detection probability, and *s* models background detections from ambient light and dark count^18^.

The free-running SPAD records photon detections over a time interval [0, *T*] discretized into uniform bins of size Δ, where *T* is the inverse of the laser repetition rate. Let *h*_*ijk*_ denote the accumulated counts in bin *k* over the *N* pulses and ${\lambda }_{ijk}({\alpha }_{ij},{z}_{ij},s)={\int }_{k{\rm{\Delta }}}^{(k+\mathrm{1)}{\rm{\Delta }}}\,{r}_{ij}(t,{\alpha }_{ij},{z}_{ij},s)dt$ the Poisson rate for the number of detections in each bin *k*. The proposed probabilistic reconstruction method infers the latent variables ***z***, ***α*** using maximum-a-posteriori (MAP) estimation, which relies on the probability *P*(**h**_*ij*_|**λ**_*ij*_) of observing a histogram **h**_*ij*_ ∈ **Z**^*T*^ given means **λ**_*ij*_(*α*_*ij*_, *z*_*ij*_, *s*) ∈ **R**^*T*^ and priors on depth and albedo.

Prior work on 3D imaging using SPAD detectors focuses on the low-flux regime^18,27,31,32^ in which the expected number of photon detections per pulse is significantly smaller than one. In the low-flux regime we may neglect dead time and approximate the observations *h*_*ijk*_ as being conditionally independent across bins *k*, with corresponding Poisson probability mass function *P*(*h*_*ijk*_|**λ**_*ij*_). For medium and high-flux measurements the conditional independence approximation breaks down because dead time ensures that at most one photon detection is recorded per pulse^25^. The probability of an aggregated count in a single histogram bin, taking dead time into account, is given by the multinomial distribution

3 $$P({{\bf{h}}}_{ij}|{{\boldsymbol{\lambda }}}_{ij})=\frac{N!\exp {(-{{\bf{1}}}^{T}{{\boldsymbol{\lambda }}}_{ij})}^{N-{{\bf{1}}}^{T}{{\bf{h}}}_{ij}}}{{h}_{ij1}!\cdots {h}_{ijT}!(N-{{\bf{1}}}^{T}{{\bf{h}}}_{ij})!}\prod _{\ell \mathrm{=1}}^{T}{(\exp (-\sum _{k\mathrm{=1}}^{\ell -1}{\lambda }_{ijk})-\exp (-\sum _{k\mathrm{=1}}^{\ell }{\lambda }_{ijk}))}^{{h}_{ij\ell }},$$

where **1** is the vector of all ones. We refer to the Supplemental Methods for a detailed derivation of this probabilistic model. The observation model holds across the full measurement range, from low-flux to high-flux regimes.

### Reconstruction algorithm

After scanning a scene, temporal histograms **h**_*ij*_ are available for each point (*i*, *j*). To reconstruct scene reflectance and depth from these histograms, we find the MAP estimate of ***α*** and ***z***, along with the ambient term *s*, using the observation model and the prior assumption that the gradients of reflectivity and depth maps are sparse. Inspired by prior work on depth and reflectivity estimation^18,27,28^, we place transverse anisotropic total variation (TV) priors directly on ***α*** and ***z***. The MAP estimate is given by solving the optimization problem

4 $$\begin{array}{c}{\rm{minimize}}\\ {\boldsymbol{\alpha }},{\bf{z}},s\end{array}\,\sum _{i=1}^{m}\sum _{j=1}^{n}\,-\,\mathrm{log}\,P({{\bf{h}}}_{ij}|{{\boldsymbol{\lambda }}}_{ij}({\alpha }_{ij},{z}_{ij},s))+{\gamma }_{1}{\Vert \nabla {\boldsymbol{\alpha }}\Vert }_{1}+{\gamma }_{2}{\Vert \nabla {\bf{z}}\Vert }_{1},$$

where ***α*** and ***z*** are the unknown variables, and ∇ is the gradient operator. To solve this problem, we develop a proximal algorithm for this non-convex optimization problem that decouples the sparsity-promoting prior terms from the likelihood term. Specifically, we minimize the joint objective by introducing slack variables **v**^***α***^, **v**^***z***^, **v**^*s*^ for albedo, depth and ambient terms. We then optimize each unknown term in an alternating fashion (see Supplemental Material for details). As a result of this proximal optimization scheme, the log-likelihood minimization becomes separable in the measurement *ij* as

5 $$\begin{array}{c}{\rm{minimize}}\\ {\boldsymbol{\alpha }},{\rm{z}},s\end{array}\,-\mathrm{log}\,P({{\bf{h}}}_{ij}|{{\boldsymbol{\lambda }}}_{ij}({\boldsymbol{\alpha }},{\rm{z}},s))+\frac{\xi }{2}{({\bf{z}}-{{\bf{v}}}^{{\bf{z}}})}^{2}+\frac{\xi }{2}{({\boldsymbol{\alpha }}-{{\bf{v}}}^{{\boldsymbol{\alpha }}})}^{2}+\frac{\xi }{2}{(s-{{\bf{v}}}^{s})}^{2},$$

which is an optimization problem over a non-linear tri-variate loss function and quadratic proximal closeness terms with scalar weight *ξ*. We solve the minimization problem with per-pixel parallel Newton Methods, as derived in the Supplemental Methods.

This reconstruction method improves prior SPAD depth imaging techniques both by accounting for dead time in the observation model, which is crucial for high and medium-flux scenarios, and by jointly estimating ***α*** and ***z*** directly from the raw histogram data. Previous approaches, on the other hand, apply a sequence of transformations to the data, estimating ***α*** and ***z*** in separate stages^18,27,31,32^, which limits the reconstruction performance.

### Experimental validation

**Figure 2:**
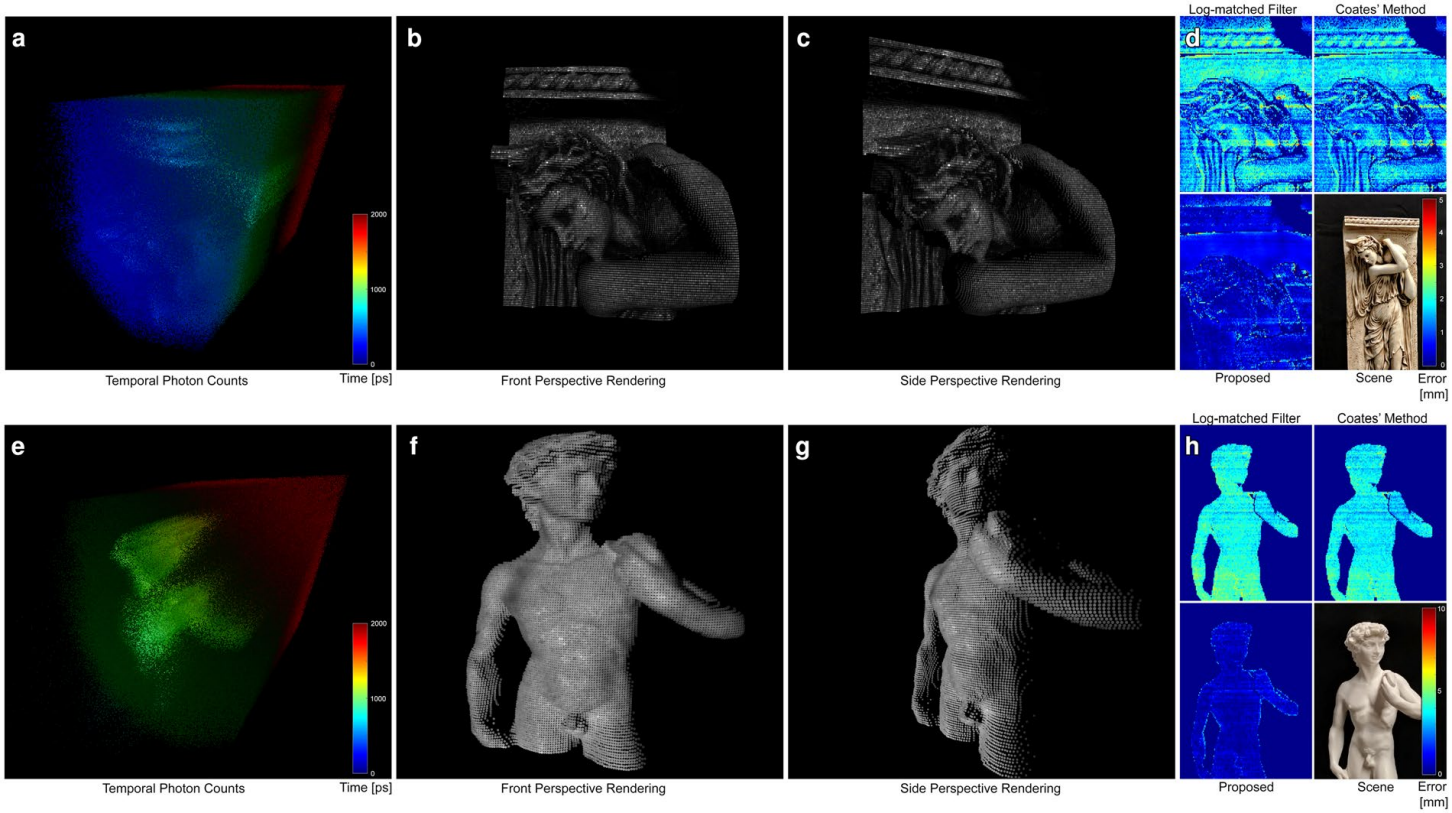
Experimental reconstructions. A recorded spatio-temporal distribution of photon counts **(a**,**e)** is processed to estimate a 3D point cloud **(b**,**c**,**f**,**g)** that contains both depth and albedo information, here shown for two different scenes (photographs shown in **(d**,**h)**). The color-coded errors maps **(d**,**h)** directly compare the results of several depth estimation techniques, including log-matched filtering^32^, Coates’ method^25^ followed by Gaussian fit (on high-flux measurement), and the proposed method.

We evaluate the proposed method on measurements experimentally acquired with the setup illustrated in Fig. 1. In Fig. 2, we assess the performance of the proposed method on two scenes with highly varying reflectance and depth profiles. Both scenes contain objects with complex geometries and varying reflectance properties, including specular behavior for the “Statue of David” scene and Lambertian reflectance with spatially varying albedo in the “Bas-relief” scene. For both scenes, we capture a ground truth reference measurement with a 5% Neutral Density filter in the laser path which eliminates pileup distortions by damping the source intensity. To minimize shot noise fluctuations at the low count rates, we acquire very long sequences of 6 s length per spot at 4 MHz laser repetition rate. We scan every scene at a spatial resolution of 150 × 150 points. Hence, a full reference measurement is acquired in 150 ⋅ 150 ⋅ 6 s = 37.5 h per scene. The ground truth depth is extracted from long-exposure measurements using log-matched filtering^32^ with the impulse response calibrated using a planar target captured under the same acquisition settings, i.e. unaffected by pileup. The experimental measurements, which serves as input for the proposed method, are acquired without any filters in the optical path, please see the Supplemental Material for additional details.

For each scene in Fig. 2, depth and albedo reconstructions along with the corresponding error maps are shown. We show perspective renderings of the point cloud reconstructions for two different viewpoints. These results verify that the proposed method achieves high-quality depth and albedo reconstructions unaffected by scene-dependent pileup or shot-noise distortions. Specifically, we compare our approach against the conventional log-matched filter estimate^32^ as well as Coates’ pileup correction method^25^ followed by a Gaussian fit. The effect of pileup becomes apparent in the error map for the log-matched filter estimate resulting in more than 5 mm depth error for the “Statue of David” scene. Existing methods do not effectively suppress pileup and therefore suffer from strongly scene-dependent depth precision. In contrast, our method achieves sub-picosecond accuracy independent of scene depth and reflectance, despite the FWHM of the laser pulses being longer than 50 ps. We include additional comparisons in the Supplemental Results where we also evaluate the proposed approach in the low-flux regime and demonstrate that our probabilistic method achieves close to an order of magnitude increased temporal resolution.

**Figure 3:**
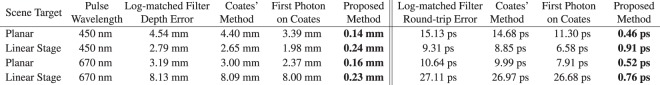
Experimental validation of sub-picosecond accuracy on recorded single-pixel data without spatial priors. The average depth and round-trip time error for two scenes are shown, for the 450 nm Alphalas LD-450-50 laser (FWHM of 90 ps) and the 670 nm Alphalas LD-670-50 laser (FWHM of 50 ps), respectively. The background level is 5% for all scenes. We compare reconstructions of the conventional log-matched filter estimate^32^, Coates’ method^25^ followed by a Gaussian fit, Shin *et al*.^18^ on Coates-corrected measurements, and the proposed method.

Next, we validate the sub-picosecond accuracy of the proposed approach without using spatial priors, i.e. relying solely on the probabilistic pileup model for individual histogram measurements. Specifically, we acquire a sequence of single-point measurements of a planar Lambertian target which is moved along the optical axis using a linear motion stage for 100 measurement points uniformly spaced with 0.5 mm distance. The setups for this measurement scenario and for an additional horizontal scanline measurement are shown in the Supplemental Information. In addition to the conventional log-matched filter estimate^32^ and Coates’ method^25^, we compare the proposed method against and Shin *et al*.’s method^18^ applied on the Coates-corrected histogram data, which adds censoring and background signal suppression^18^ to Coates’ method. Figure 3 shows the average absolute error in depth and round-trip time for both of these measurements. The results demonstrate that, even without spatial priors, the proposed probabilistic method outperforms existing state-of-the-art approaches by more than an order of magnitude. Adding the proposed spatial prior reduces temporal error by a factor of 2× on average. We refer to the Supplemental Results for additional experimental results and extensive evaluation in simulation.

### Optimal regime of photon counts

**Figure 4:**
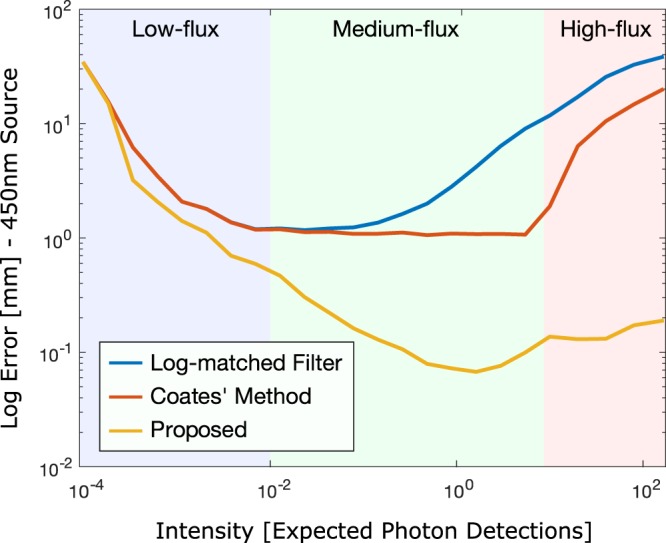
Optimal photon count regime. Depth reconstruction accuracy for varying photon counts for the 450 nm Alphalas LD-450-50 laser (FWHM of 90 ps). The conventional log-matched filter, Coates’ method^25^, and the proposed method are compared. The optimal number of photon counts lies around the unconventional region of 1 photon detected per pulse on average, independent of the impulse response and for a broad range of histogram bin widths, see Supplemental Results.

Next, we analyze the performance of the proposed method with a fixed dwell or exposure time for a varying incident photon flux. The proposed probabilistic method achieves optimal accuracy in an unconventional, pileup-affected regime with around 1 photon detection per pulse (see Fig. 4). This plot is generated in simulation without the use of spatial priors for the 450 nm Alphalas LD-450-50 laser with *N* = 10^4^ shots. As expected, the log-matched filtering approach performs best when the measurements are neither too noisy nor too much affected by pileup. Existing sequential pileup correction methods, such as Coates’ method^25^, alleviate pileup and effectively extend the range where optimal performance can be reached beyond 1 expected photon detection per pulse. The accuracy of the proposed method is identical to previous methods for the low-flux regime, because we only consider a single pixel and no spatial priors are used. As the photon count increases, our approach substantially improves upon all existing methods by up to two orders of magnitude. Optimal precision is achieved for expected photon counts around 1 per pulse, which is significantly higher than the low-flux regime in which existing methods operate. These results motivate an optimal photon flux regime for 3D imaging that is far outside the conventional low-flux regime, see Supplemental Results for additional discussion. In the Supplemental Results, we also analyze the effect of the histogram bin width on timing accuracy in this optimal flux regime. The accuracy is consistent across a broad range of histogram bin-widths from sub-2 ps up to 80 ps, demonstrating that the proposed approach not only improves on the state-of-the-art by more than an order of magnitude in accuracy, but also reduces the timing resolution requirements on the detector side.

## Discussion

Despite laser pulse widths being larger than 50 ps FWHM, the proposed probabilistic image formation model and corresponding inverse methods achieve sub-picosecond accuracy for active 3D imaging. Moreover, the proposed method achieve this precision across a wide dynamic range, from low-flux to high-flux measurements. These capabilities are achieved by accurately modeling pileup distortions in the image formation model and by solving the inverse problem jointly for all latent variables using statistical priors. In the future, the proposed method could also facilitate long-range acquisition without distance-dependent reduction in repetition rate, by multiplexing multiple pileup-affected responses into the same histogram and unwrapping the pileup-corrected peaks. The proposed method paves the way for fast and precise photon-efficient 3D imaging systems in practical scenarios where widely-varying photon counts are observed in a scene. Applications across disciplines may significantly benefit from these capabilities, including 3D mapping and navigation, autonomous driving, robotic and machine vision, geographic information science, industrial imaging, and microscopic imaging.

## Methods

### Equipment details

The hardware setup consists of a time-resolved sensor, pulsed laser, illumination and collection optics, and a set of scanning mirrors to achieve a raster scan illumination pattern. The sensor is a PDM SPAD from Micro Photon Devices with a 100 × 100 μm sensor area, 27 ps timing jitter (measured with a 100 kHz laser at 675 nm), and 40.9 dark counts per second. Timing of photon arrivals is captured with a PicoHarp 300 Time-Correlated Single Photon Counting (TCSPC) module. The illumination source is a 450 nm or 670 nm picosecond laser (ALPHALAS PICOPOWER-LD-450-50, PICOPOWER-LD-670-50). The 450 nm and 670 nm versions have pulse widths of 90 ps and 50 ps and average power of 0.406 mW and 0.11 mW respectively at a 10 MHz pulse repetition rate. The collection optics are designed to extend the field of view of the SPAD across the area scanned by the illumination source and consist of a 75 mm objective lens (Thorlabs AC508-075-A-ML), a 30 mm relay lens (Thorlabs AC254-030-A-ML) and a microscope objective (Olympus UPLFLN 20× objective). The laser spot is minified using a 50 mm (Thorlabs AC254-050-A-ML) and 250 mm (Thorlabs AC254-250-A-ML) lens relay and scanned with mirrors driven by a two-axis galvanometer (Thorlabs GVS012).

### Calibration details

All parameters of the image formation model are described in the observation model section. The calibration of these parameters is performed once for a given laser, i.e., pulse shape, and detector configuration. The detector’s photon detection probability *μ* = 0.34 at 450 nm (*μ* = 0.33 at 670 nm) and dark count rate *d* = 40.9 *c*/*s* were calibrated using the method of Polyakov^33^. The remaining unknowns are {*a*_*k*_, *b*_*k*_, *c*_*k*_|*k* ∈ 1, …, *K*} for the Gaussian mixture model $\tilde{g}$ we use *K* = 8 and calibrate for the 450 nm Alphalas LD-450-50 laser the values {(0.57, 220.4, 32.0), (0.0059, 203.8, 10.2), (0.003, 214.6, 18.7), (−0.57, 220.3, 32.0), (0.003, 255.6, 47.5), (0.002, 297.9, 67.4), (0.009, 199, 7.1), (0.0003, 357.4, 143.2)}, and for 670 nm Alphalas LD-670-50 laser the values {(0.04, 199.4, 6.4), (0.004, 206.5, 7.0), (−0.001, 187.9, 12.5), (0.005, 204.8, 17.8), (0.003, 225.1, 27.2), (0.002, 254.6, 42.3), (0.0008, 301.0, 69.3), (0.0003, 388.7, 136.0)}. Measurements of a scene consisting of a single diffuse reflector placed at 1 m distance are acquired to estimate these parameters. Specifically, an ND filter with 1% transmission is placed in the illumination path, and a low-noise histogram of a single point reflector is accumulated with *N* = 10^9^ pulses, requiring approximately 17 minutes at a 1 MHz laser repetition rate. The high-absorption ND filter ensures a low-flux regime where pileup can be ignored and where the histogram counts are Poisson distributed. For the high number of 10^9^ shots, these histogram measurements can be well approximated by a Gaussian distribution. Finally, the mixture parameters are estimated using efficient, conventional expectation-maximization algorithms^34^. Please see the Supplemental Methods for calibration results for the for the 450 nm Alphalas LD-450-50 laser and the 670 nm Alphalas LD-670-50 laser.

### Algorithm parameters

The algorithm hyper-parameters are *γ*_1_ = *γ*_2_ = 10^−1^. These values were determined using simulations. Initial values of ***α*** = 1, ***z*** = 0 are used for the reconstruction algorithm in the algorithm section. In practice, warm-starting with the log-matched filter or Coates’ estimate reduces the iterations needed to converge. The termination criteria for the proposed non-convex optimization algorithm are detailed in the Supplemental Methods. The algorithm run time with unoptimized Matlab code averages around 100 s on a Intel i7 2.6 GHz notebook computer. Note that the likelihood proximal operator from Eq. (5), which dominates the algorithm runtime, is embarrassingly parallel across all measurement points, and hence is suited for implementation on modern GPU hardware. Specifically, the operator is parallelizable into 150 ⋅ 150 = 22500 independent threads for the proposed setup configuration, promising run times on the order of a few milliseconds in future implementations, see Supplemental Material.

## Electronic supplementary material

Supplementary Information

## Data Availability

The code and data used to generate the findings of this study are available on GitHub: https://github.com/computational-imaging/spad_pileup.
